# Human capital development and economic sustainability linkage in Sub-Saharan African countries: Novel evidence from augmented mean group approach

**DOI:** 10.1016/j.heliyon.2024.e24323

**Published:** 2024-01-09

**Authors:** Mulugeta Bekele, Maria Sassi, Kedir Jemal, Beyan Ahmed

**Affiliations:** a Agricultural Economics, Haramaya University, Ethiopia; b Agricultural Economics, University of Pavia, Italy

**Keywords:** Human capital development, Economic sustainability, Quality education, Augmented mean group, Sub-Saharan Africa

## Abstract

Sustainable Development Goal number four (SDG-4) strongly emphasizes quality education, which is crucial to human capital development. The importance of human capital development for sustainable economic development has thus risen to the top of the global policy agenda. However, the empirical literature on the topic has several limitations, including utilizing inappropriate measurement of human capital development and being unable to investigate the quality dimensions of education. Therefore, this study used years of schooling and return on education to fill the gap, and also considered the role of institutional and political factors in its empirical estimation. The main objective of this study is to investigate the effect of human capital development on economic sustainability in 30 Sub-Saharan African countries, employing panel data from 2000 to 2020. The augmented mean group model was used in the empirical investigation. The findings of the study showed that human capital development negatively and significantly affects economic sustainability in Sub-Saharan African countries. Thus, among the policy alternatives that Sub-Saharan African countries and policymakers should pursue to attain the goals of economic sustainability is revisiting the entire human capital development architecture and putting more of an emphasis on quality education than on access to education.

## 1 Introduction

In line with Sustainable Development Goal eight (SDG-8), which places a strong emphasis on both decent work and economic growth, Sub-Saharan Africa (SSA) countries are primarily expected to meet an economic growth target of at least 7% annually [1]. However, though substantial economic progress was made over the past few decades, Sub-Saharan Africa (SSA) has struggled to achieve sustainable economic development, putting the Sustainable Development Goals (SDGs) viability into doubt due to the developing character of SSA member states, especially those that are struggling with severe food insecurity, inadequate infrastructure, income inequality, and a lack of capital [2,3].

The literature on endogenous growth theory has inspired empirical studies on the relationship between human capital development and the growth rate of real output [4]. Thus, human capital development, which requires a long-term investment, is necessary for the emergence and sustenance of a knowledge-based economy that relies on creating, distributing, and using knowledge and information [5]. This type of economy increasingly relies on the efficient use of intangible assets, such as knowledge, skills, and innovative potentials, as the primary source of competitive advantage. According to several pieces of research [6,7], human capital development is a priceless way to achieve and maintain a higher consumption trajectory in the future.

When fostering engagement in sustainable economic activities, the pivotal role of human capital, particularly education, cannot be overstated [8,9,10]. Individuals endowed with higher levels of human capital exhibit a greater inclination towards environmentally friendly products and lifestyles [11,12]. The acquisition of knowledge regarding sustainable practices and the impact of human actions on the natural world empowers individuals to effect positive changes. This comprehensive awareness fosters eco-friendly habits and instils a propensity for long-term decision-making across diverse economic spheres [13,14,15]. Furthermore, investing in human capital serves as a proactive measure to avert long-term ecological damage [8,16].

However, for the past four decades, the SSA region has been a major source of large-scale human capital flight and brain drain [2]. For instance, according to Ref. [17], one-third of the most educated people in the region are thought to live outside of the region, primarily in Western Europe and North America, where earnings are higher. On the other hand, because they lacked the skills that the labour market demanded, as well as due to longer and more unstable school-to-work transitions, greater labour market inequalities and informalities, and a greater detachment from the labor market, young graduates in the region were particularly vulnerable to severe unemployment [18]. These elements significantly affect the region's human capital development or return on investment in education, rendering educational investments counterproductive.

Furthermore, in SSA, quality education, which is strongly highlighted by Sustainable Development Goal number 4, depends on foreign development assistance for its financing. Since 1990, more than 500 billion USD has been spent on education-related aid, most of it going to low-income countries, particularly those in Sub-Saharan Africa [19]. However, except for a few, a disproportionate number of states have been unable to change their educational system and continue to rely too heavily on successive rounds of foreign aid to close the funding gaps. The infinite do loop, in which inadequate aid leads to the need for additional aid, will persist until serious actions extending to curriculum development are undertaken. Accordingly, on the basis of data demonstrating that a sizable portion of young people are failing to meet the minimum learning standards, the World Bank recently declared a “learning crisis” [20].

According to an empirical study by Ref. [21], human capital development is critical in creating sustainable behaviors that promote sustainable development. These practices encompass a spectrum of practices, including investing in renewable energy sources, implementing sustainable transportation systems, implementing energy-efficient technologies, and increasing climate change awareness and education. It also improves resource efficiency and sustainability [22,10]. Human capital promotes sustainability by increasing participation in environmentally friendly activities such as using renewable energy [23,24]. Additionally, it improves the quality of the abatement action [25,21].

Prior empirical studies on the subject indicated a positive relationship between human capital development and economic growth or development [[26], [27], [28], [29], [30], [31], [32], [33]]. According to Ref. [34], encouraging green growth necessitates increasing the accumulation of innovative human capital. However, the majority of the empirical research made the common mistake of relying on access to education measurements rather than quality education aspects as a measure of human capital development. For instance, they used government expenditure on health and education [[30], [31], [32], [33], [35]] and enrolment rates [26,31,32], as a proxy for human capital development, which this study believes as a critical gap in the literature.

Therefore, the novelty of this study lies in its use of the human capital index, a measure of human capital development based on return on education and years of schooling, which is a relatively appropriate measure that better explains educational quality. Further, this study considered the role of institutional and political factors in its estimation, which is vital for developing countries like SSA but typically ignored in earlier studies [27,28,[30], [31], [32], [33]]. Moreover, this study controlled for the possible effect of key variables like natural resource endowment, domestic investment, government expenditure, financial development and policy dummy.

Thus, this study is designed to investigate the effect of human capital development on economic sustainability in Sub-Saharan Africa from 2000 to 2020.

## 2 Methodology

### 2.1 Theoretical framework and empirical model specification

The theoretical framework in this study is in line with endogenous growth theory, which describes the long-run growth rate of real output at a rate determined by forces internal to the economic system, particularly those governing the opportunities and incentives for the development of technological knowledge [36]. Further, the study followed [35] specification to look into the impact of human capital development on economic sustainability as follows:

(1) $${ANNI}_{it}=\alpha_{it}+\beta_{1}{HCI}_{it}+\beta_{2}Z_{it}+\varepsilon_{it}$$

where ${ANNI}_{it}$ represents adjusted net national income (a measure of economic sustainability) for country i at year *t*, ${HCI}_{it}$ stands for human capital index (a measure of human capital development), $Z_{it}$ denotes a vector of control variables for economic sustainability, $\alpha_{it}$ is the constant, $\beta_{1}$ and $\beta_{2}$ shows the slope coefficients and $\varepsilon_{it}$ is the error term. The selection of $Z_{it}$ (vector of control variables) in this study is informed by either theoretical or empirical literature.

The empirical model adopted in this study follows this specification, which links human capital development and economic sustainability, including the vector of control variables that affect economic sustainability, as follows:

(2) $${ANNI}_{it}=\alpha_{it}+\beta_{1}{HCI}_{it}+\beta_{2}{TNRR}_{it}+\beta_{3}{GCF}_{it}+\beta_{4}{GE}_{it}+\beta_{5}{BM}_{it}+\beta_{6}{IQ}_{it}+\beta_{7}YD+\varepsilon_{it}$$

where the vector of control variables includes total natural resource rent, TNRR, as a measure of natural resource endowment, gross capital formation, GCF, as a measure of domestic investment, government expenditure (GE), broad money, BM, as a measure of financial development, institutional quality (IQ) and a policy dummy (PD). The notations i and t respectively demonstrate country and year. Further, $\alpha_{it}$ is the constant, $\beta_{1},\beta_{2},\beta_{3},\beta_{4},\beta_{5},\beta_{6},and\;\beta_{7}$ shows the slope coefficients and $\varepsilon_{it}$ is the error term.

The dependent variable in this study is economic sustainability. It is defined as the development that is concerned with the improvement of the living standards of people by providing lasting and secured livelihood minimizing resource depletion and environmental degradation [37,38]. In line with the theoretical definition provided, this study used adjusted net national income per capita as a proxy for economic sustainability. Thus, adjusted net national income is calculated as GNI minus consumption of fixed capital and natural resources depletion, and it is expressed in hundreds (current USD). This study addressed the gap in previous empirical studies [39,40], they used GDP per capita growth as a measure of economic sustainability, which is calculated without making deductions for depreciation of fabricated assets or depletion and degradation of natural resources.

The following is a discussion of the explanatory variables used in this study. Human capital development is measured by the human capital index, calculated based on years of schooling and returns to education. Natural resource endowment is measured by total natural resources rents as a percentage of GDP. Total natural resources rents are the sum of oil rents, natural gas rents, coal rents, mineral rents, and forest rents. Domestic investment is measured by the per cent annual growth rate of gross capital formation at constant local currency. Government expenditure is measured by the general government's final consumption expenditure as a percentage of GDP. Financial Development is measured by broad money as a percentage of GDP. The institutional quality is an index based on six indicators, i.e., voice and accountability (VA), government effectiveness (GE), control of corruption (CC), political stability and absence of violence/terrorism (PSAV), regulatory quality (RQ), and the rule of law (RL). The policy dummy takes the value 0 from 2000 to 2015 during the Millennium Development Goals (MDGs) and 1 from 2016 to 2020 during the Sustainable Development Goals (SDGs). The SDGs and economic sustainability are closely interconnected. They emphasize the importance of adopting sustainable practices in various sectors, including human capital development.

Except for the data on institutional quality, which is from the World Governance Indicators data set accessible at https://databank.worldbank.org/source/worldwide-governance-indicators, and human capital index, which is from Penn World Table database available at https://www.rug.nl/ggdc/productivity/pwt/?lang=en, all of the variable data were sourced from the World Bank's World Development Indicators data set, which is openly accessible at https://data.worldbank.org/.

The scope of the study was impacted by data available from 2000 to 2020, which included the following 30 Sub-Saharan African countries: Angola, Benin, Botswana, Burkina Faso, Burundi, Cape Verde, Cameroon, Central African Republic, Chad, Comoros, Congo, Democratic Republic of Congo, Ethiopia, Gambia, Ghana, Ivory Coast, Kenya, Madagascar, Malawi, Mali, Mozambique, Namibia, Niger, Nigeria, Senegal, South Africa, Sudan, Tanzania, Togo, and Zambia. Because this study covered a sufficiently large sample of countries from the Eastern, Western, Central, and Southern parts of the region, its conclusions can represent the whole Sub-Saharan Africa region.

### 2.2 Econometric technique

All studies involving time-dependent data in the panel undergo a series of estimation processes to attain the intended outcomes. Based on this, the study's analytical methodology incorporated the panel econometric techniques listed below. The study began by testing for cross-sectional dependency and slope homogeneity among the variables using the [41,42] tests, respectively. In the second stage, the stationarity qualities of the study variables are examined using the CIPS and CADF panel unit root test methodologies. Following proof of an exclusive order of stationarity, the [35] panel co-integration test was used to look for co-integration relationships between variables. The long-term coefficients were then estimated using the panel-augmented mean group technique.

#### 2.2.1 Cross-sectional dependence test

Panel data can be impacted by cross-sectional dependence, which occurs when all units within a cross-section are correlated. According to Ref. [41], this condition is frequently linked to the influence of some unobserved common factors, spatial effects, and spillover effects from socioeconomic relationships. Testing for cross-sectional dependence is thus crucial in panel data analysis because its presence may result in inconsistent estimates and misleading information [41,43].

Although the empirical literature also mentions the [44] Lagrange Multiplier (LM) test, it might be inconsistent and inappropriate when the number of cross-sectional units is larger than the number of time periods. Thus, this study preferred using the Cross-Sectional Dependence (CD) test proposed by Ref. [41] to detect this possibility because there are 30 cross-sectional units/countries and 21 time periods/years in this study.

Accordingly, Pesaran (2004) provided the CD test to adjust the bias in the LM test as follows:

(3) $$\text{CD}=\sqrt{\frac{2T}{N\left(N-1\right)}}\;\sum_{i=1}^{N-1}\sum_{j=i+1}^{N}\frac{\left(T-k\right){\hat{\rho_{\text{ij}}}}^{2}-E\left[\left(T-k\right){\hat{\rho_{\text{ij}}}}^{2}\right]}{\text{Var}\left[\left(T-k\right){\hat{\rho_{\text{ij}}}}^{2}\right]}$$

where N is the sample size, T set for the time period, k denotes the identity matrix, and $\hat{\rho_{ij}}$ denotes the pairwise correlation coefficient obtained from OLS estimation for each cross-section dimension, i, given j=i+1. Thus, the CD statistic is tested against the null hypothesis of no cross-sectional dependence.

#### 2.2.2 Slope homogeneity test

All cross-sectional units/countries are assumed to share the same model parameters in homogeneous panel data models. Heterogeneous models, however, allow for individual variances in some or all of the model parameters. In light of this, slope homogeneity can produce unreliable and untrustworthy estimates if the panel data is heterogeneous. Thus, this study employed the Swamy test method suggested by Ref. [42] to evaluate the slope homogeneity phenomena as indicated in equations (4), (5), (6).

(4) $$\bar{\nabla }=\sqrt{N}\left[\frac{N^{-1}\overset{ˇ}{S}-k}{\sqrt{2k}}\right]$$

(5) $$\bar{\nabla }\text{adj}=\sqrt{N}\left[\frac{N^{-1}\overset{ˇ}{S}-k}{\sqrt{\frac{2k\left(T-k-1\right)}{T-1}}}\right]$$

(6) $$\overset{ˇ}{S}=\sum_{i=1}^{N}{\left(\hat{\beta_{i}}-{\tilde{\beta }}_{\text{WFE}}\right)}^{\prime }\frac{X_{i}^{\prime }M_{\tau }X_{i}}{{\overset{ˇ}{\delta }}^{2}}\left(\hat{\beta_{i}}-{\tilde{\beta }}_{\text{WFE}}\right)$$

$\bar{\nabla }$, $\bar{\nabla }\text{adj}$ and $\overset{ˇ}{S}$ stands for the standardized dispersion, the biased-adjusted statistics and the Swamy statistic (based on the dispersion of individual slope estimates), respectively. $\hat{\beta_{i}}$, refers to the pooled OLS regression coefficients for each country i ranging from 1 to N, and ${\tilde{\beta }}_{\text{WFE}\;}$ set for the weighted fixed effect (WFE) pooled estimator. Additionally, $M_{\tau }$, ${\overset{ˇ}{\delta }}^{2}$ and k are, respectively, the identity matrix, the estimate of ${\sigma_{i}}^{2}$ and the number of explanatory variables. Accordingly, the Swamy statistic value has been tested against the null hypothesis of slope homogeneity.

#### 2.2.3 Panel unit root test

Levin-Lin Chu (LLC), Im-Pesaran-Shin (IPS), augmented Dickey-Fuller (ADF), and Phillips-Perron (PP) are some of the first-generation panel unit root tests that are invalidated by cross-sectional dependence [41]. As a result [41], second-generation panel unit root tests, which are trustworthy in the presence of cross-sectional dependence, were applied in this study. These tests include the cross-sectional augmented Dickey-Fuller (CADF) and the cross-sectional augmented Im-Pesaran-Shin (CIPS). A formula for calculating the CADF statistic is provided below:

(7) $$\Delta y_{i,t}=\alpha_{i}+\beta_{i}y_{i,t-1}+\gamma_{i}{\bar{y}}_{t-1}+\delta_{i}\Delta {\bar{y}}_{i,t}+\varepsilon_{\text{it}}$$

where ${\;\bar{y}}_{t-1}$ and $\Delta {\bar{y}}_{i,t}$ respectively denotes the cross-sectional averages of lagged levels and the first differences of individual series.

(8) $${\;\bar{y}}_{t-1}=\frac{1}{N}\sum_{i=1}^{N}y_{i,t-1}$$

(9) $$\Delta {\bar{y}}_{i,t}=\frac{1}{N}\sum_{i=1}^{N}\Delta y_{i,t}$$

The CADF statistic has been computed by averaging the ${\text{CADF}}_{i}$ as follows:

(10) $$\text{CIPS}=\frac{1}{N}\sum_{i=1}^{N}{\text{CADF}}_{i}$$

CADFi, however, is the t-statistic of the CADF regression as defined by equation (7).

Accordingly, the variables can be considered stationary if the absolute values of CIPS and CADF statistics are greater than the critical values at a 5% level of significance.

#### 2.2.4 Panel Co-integration test

The panel co-integration test developed by Ref. [45] was used in this investigation because it is robust in the presence of cross-sectional dependence. It is based on the error-correction model described below:

(11) $$\Delta y_{i,t}=\delta_{i}^{\prime }d_{t}+\rho_{i}\left(y_{i,t-1}-{\beta_{i}^{\prime }X}_{i,t-1}\right)+\sum_{j=1}^{k}\varphi_{\text{ij}}y_{i,t-j}+\sum_{j=1}^{k}\varphi_{\text{ij}}X_{i,t-j}+\mu_{i,t}$$

in equation (11), $\rho_{i}$ is the adjustment term that indicates how quickly the system adjusts back to equilibrium.

The test is built on the least-squares estimates of $\rho_{i}$ with the null hypothesis assuming no co-integration. Accordingly, the group mean statistics have been computed as follows:

(12) $$G\tau =\frac{1}{N}\sum_{i=1}^{N}\frac{\rho_{i}\;}{\text{Se}\left(\hat{\;\rho_{i}}\right)}$$

(13) $$G\alpha =\frac{1}{N}\sum_{i=1}^{N}\frac{T\rho_{i}\;}{\rho_{i}^{\prime }\left(1\right)}$$

It can be inferred that co-integration exists in at least one cross-sectional unit of the panel when Gτ and Gα statistics reject the null hypothesis.

In the meanwhile, the formulas below are used to extract the panel statistics:

(14) $$P\tau =\frac{\hat{\;\rho_{i}}\;}{\text{Se}\left(\hat{\;\rho_{i}}\right)}$$

(15) $$P\alpha =T\hat{\;\rho }$$

It is possible to conclude that co-integration exists in the whole panel if the null hypothesis is rejected.

#### 2.2.5 Panel long-run estimates

According to Refs. [[46], [47], [48]], conventional panel regression procedures may be inconsistent and biased in the presence of cross-sectional dependency. In this study, the AMG estimator developed by Ref. [49] is employed since it is particularly robust to cross-sectional dependency and parameter heterogeneity. The AMG method is critical because it may be used on models with different slopes. Despite these flaws, the test yields reliabl [48]e results even in the presence of cross-sectional dependency, non-stationarity, and endogeneity.

The common dynamic effect parameter in equation (16) specifies the unobservable common factors $\left(f_{t}\right)$ that the AMG estimator captures. Refer to the first difference OLS equation below to illustrate the AMG estimator:

(16) $${\Delta y}_{\text{it}}=\alpha_{i}+\beta_{i}{\Delta X}_{\text{it}}+\sum_{t=1}^{T}\theta_{t}D_{t}+\varphi_{i}f_{t}+\varepsilon_{\text{it}}$$

where $y_{\text{it}}$ and $X_{\text{it}}$ are variables; $\beta_{i}$ refers the country-specific slope; $f_{t}$ denotes the unobserved common factor with heterogeneous factor; $\alpha_{i}$ and $\varepsilon_{it}$ are the intercept and error terms, respectively. Further, Δ set for the first-difference operator, $\beta_{i}$ for country-specific coefficients and $\theta_{t}$ for time-specific coefficients.

Then, using the across-panel averaged group-specific parameters, the AMG estimator is obtained:

(17) $$\text{AMG}=\frac{1}{N}\sum_{i=1}^{N}\tilde{\beta_{i}}$$

in equation (17), $\tilde{\beta_{i}}$ set for the estimator of $\beta_{i}$ in equation (16). This study employed the AMG approach to estimate the long-run parameters because its performance in Monte Carlo simulation is unbiased and efficient for different N (number of observations) and T (time) settings.

## 3 Results and discussion

The empirical findings of the study are presented and discussed in this section. Data analysis was conducted using STATA 15 software. In the subsections below, both descriptive and econometric results are discussed.

### 3.1 Descriptive statistics

To explain the type of data used for the empirical analysis, several important descriptive statistics are presented in this section. All of the statistics were estimated using observations from the study targets, which were drawn from 30 SSA countries and covered 2000–2020.

The descriptive statistics and correlation matrix of the variables taken into consideration in this study are shown in Table 1 and Table 2, respectively. Based on the descriptive statistics presented in Tables 1 and it can be concluded that the data is stable and free of outliers. Additionally, the correlation matrix in Table 2 shows that there is no threat of either collinearity or multi-collinearity. The highest correlation coefficient between HCI and all the other explanatory variables is less than 0.8, the value used as the rule of thumb for high correlation. This result indicates that neither collinearity nor multi-collinearity is considered an issue in our data.

**Table 1:** Statistical description of the variables considered in this study.

Variable	Observation	Panel characteristics	Mean	Std. deviation	Minimum	Maximum
**ANNI**	N = 630 *n* = *30* T = 21	Overall	11.23	12.67	0.48	70.82
		Between		11.84	1.29	49.64
		Within		4.99	−15.99	32.42
**HCI**	N = 630 *n* = *30* *T* = *21*	Overall	1.70	0.41	1.01	2.93
		Between		0.40	1.17	2.72
		Within		0.13	1.25	2.21
**NRR**	N = 630 *n* = *30* *T* = *21*	Overall	10.73	10.60	0.31	58.65
		Between		9.61	0.55	42.48
		Within		4.79	−14.40	33.72
**GCF**	N = 630 *n* = *30* *T* = *21*	Overall	8.34	22.71	−65.83	231.42
		Between		6.15	−8.65	26.35
		Within		21.89	−83.84	228.01
**GFCE**	N = 630 *n* = *30* *T* = *21*	Overall	13.67	5.42	0.95	35.35
		Between		4.82	5.87	23.99
		Within		2.63	6.59	27.51
**BM**	N = 630 *n* = *30* *T* = *21*	Overall	28.99	17.11	2.86	125.30
		Between		15.93	8.78	86.21
		Within		6.86	7.15	68.08

**Table 2:** Correlation matrix.

Variable	ANNI	HCI	NRR	GCF	GFCE	BM	PD
**ANNI**	1.00						
**HCI**	0.66	1.00					
**NRR**	−0.26	−0.15	1.00				
**GCF**	−0.09	−0.12	0.06	1.00			
**GFCE**	0.43	0.18	−0.12	−0.07	1.00		
**BM**	0.62	0.36	−0.30	−0.08	0.49	1.00	
**PD**	0.11	0.18	−0.11	−0.12	0.07	0.20	1.00

### 3.2 Econometric results

Verifying if the data exhibit cross-sectional dependence is an essential initial step in the analysis of panel data. Based on the findings from Ref. [41] CD test, this study rejected the null hypothesis of no cross-sectional dependence at a 1% significance level, with the exception of domestic investment (GCF) at a 10% significance level (Table 3). Stated differently, there is substantial evidence of cross-sectional dependence in the panel data. This condition is the result of spatial effects, spillover effects from socioeconomic interactions, and some unobserved shared characteristics. These elements create inconsistent estimates. Therefore, the study considered it in the next steps of the analysis.

**Table 3:** Cross-sectional dependence test results.

Variable	CD-test	*P*-value	Correlation (mean ***ρ***)	Absolute correlation
**ANNI**	65.55***	0.000	0.686	0.723
**HCI**	91.85***	0.000	0.961	0.961
**TNRR**	20.66***	0.000	0.216	0.380
**GCF**	1.87*	0.062	0.020	0.185
**GFCE**	2.67***	0.008	0.028	0.371
**BM**	53.43***	0.000	0.559	0.614

***, * indicates significance at 1 % and 10 % level respectively. Source: Authors' calculations.

Slope heterogeneity is also present in the data, as demonstrated by the [42] test for each of the six equations, where all test statistics are highly significant at the 1% level (Table 4).

**Table 4:** Slope homogeneity test results.

Model/Equations	Statistics	Values	*P*-value
**Model-1 (IQ** = **VA)**	Delta	9.402***	0.000
	adj.	12.438***	0.000
**Model-2 (IQ** = **GE)**	Delta	8.440***	0.000
	adj.	11.165***	0.000
**Model-3 (IQ** = **CC)**	Delta	8.636***	0.000
	adj.	11.424***	0.000
**Model-4 (IQ** = **PSNV)**	Delta	9.158***	0.000
	adj.	12.115***	0.000
**Model-5 (IQ** = **RQ)**	Delta	8.248***	0.000
	adj.	10.911***	0.000
**Model-6 (IQ** = **RL)**	Delta	8.802***	0.000
	adj.	11.644***	0.000

***indicates significance at a 1 % level. Source: Authors' calculations.

Due to slope heterogeneity and cross-sectional dependence in the data, this study could not use the first-generation unit root tests for stationarity in the variables. Therefore, the study adopted CIPS and CADF test statistics, the second-generation unit root tests. Table 5 shows that some variables are stationary at the level, and some are stationary at the first difference. Meaning they are integrated at order 0 and 1, respectively, and can be referred to as I (0) and I (1). Thus, the next part examined whether these variables exhibit co-integration or the long-run linear connectivity of their paths.

**Table 5:** Second-generation panel unit-root test results.

Variable	CIPS test Statistic		CADF test Statistic		Order
	Level	First difference	Level	First difference	
**ANNI**	−1.954	−4.092***	−1.543	−2.664***	I(1)
**HCI**	−2.830***	–	−2.383***	–	I(0)
**NRR**	−2.626***	–	−2.333***	–	I(0)
**GCF**	−4.447***	–	−2.317***	–	I(0)
**GFCE**	−2.272***	–	−2.171***	–	I(0)
**BM**	−2.158***	–	−2.135 ***	–	I(0)

***indicates significance at a 1 % level. Source: Authors' calculations.

Moreover, the study detected a possible long-run relationship among the variables with the [45] co-integration. Results presented in Table 6 acknowledge the existence of co-integration among all variables in the six equations.

**Table 6:** Co-integration test results.

**Ho: No co-integration** **Ha: Some panels are co-integrated**						
**Co-integrating vector: Panel specific** **Panel means: Included** **Time trend: Not included** **AR parameter: Panel specific** **Number of panels** = **30** **Number of periods** = **21**						
	Model-1 (IQ=VA)		Model-2 (IQ = GE)		Model-3 (IQ=CC)	
	Statistic	*P*-value	Statistic	*P*-value	Statistic	*P*-value
**Variance ratio**	2.354***	0.009	2.665***	0.004	2.994***	0.001
	Model-4 (IQ=PSNV)		Model-5 (IQ = RQ)		Model-6 (IQ = RL)	
	Statistic	*P*-value	Statistic	*P*-value	Statistic	*P*-value
**Variance ratio**	2.810***	0.003	2.863***	0.002	3.028***	0.001

***indicates significance at 1 % level. Source: Authors' calculations.

Finally, the study estimated the long-run coefficients of the heterogeneous panel data using the augmented mean group (AMG) estimator after identifying cross-sectional dependence and slope heterogeneity, as well as confirming the variables' are stationary and co-integration properties. Accordingly, this study estimated six different equations using the AMG estimator, mainly to incorporate the six indicators of institutional quality separately.

The Wald test statistics also indicated significant probability statistics in all six equations that showed the robustness of the model and the long-run association among the variables. These estimates led us to test long-term coefficients, refer to Table 7.

**Table 7:** Heterogeneous parameter estimates using the Augmented Mean Group estimator results.

Variables	Model-1 (IQ=VA)		Model-2 (IQ = GE)		Model-3 (IQ=CC)	
	Coefficients	*P*-value	Coefficients	*P*-value	Coefficients	*P*-value
**HCI**	−8.353**	0.029	−4.027	0.124	−7.033***	0.006
**NRR**	−0.133***	0.000	−0.152***	0.000	−0.143***	0.000
**GCF**	0.003	0.155	0.003 *	0.053	0.003*	0.087
**GFCE**	−0.034	0.265	−0.011	0.671	−0.063**	0.020
**BM**	−0.093***	0.000	−0.098***	0.000	−0.072***	0.001
**IQ**	0.217	0.661	1.529***	0.001	0.294	0.364
**PD**	3.337***	0.000	3.186***	0.000	3.863***	0.000
**__00000R_c**	0.396***	0.000	0.376***	0.000	0.463***	0.000
**__000007_t**	0.211***	0.004	0.197***	0.008	0.276***	0.002
**_cons**	16.975***	0.002	13.689***	0.000	16.361***	0.002
**Wald chi2(7)**	58.760***	0.000	73.920***	0.000	65.680***	0.000
**Variables**	Model-4 (IQ=PSNV)		Model-5 (IQ = RQ)		Model-6 (IQ = RL)	
	Coefficients	*P*-value	Coefficients	*P*-value	Coefficients	*P*-value
**HCI**	−5.686	0.154	−7.032**	0.042	−7.921***	0.001
**NRR**	−0.144***	0.000	−0.166***	0.000	−0.165***	0.000
**GCF**	0.004**	0.024	0.004**	0.035	0.004*	0.051
**GFCE**	−0.056**	0.036	−0.063**	0.024	−0.026	0.224
**BM**	−0.065***	0.001	−0.089***	0.000	−0.074***	0.001
**IQ**	−0.267	0.261	0.283	0.303	0.600	0.163
**PD**	0.326*	0.089	2.976***	0.000	3.399***	0.000
**__00000R_c**	0.382***	0.000	0.362***	0.000	0.384***	0.000
**__000007_t**	0.195**	0.042	0.215***	0.000	0.192*	0.060
**_cons**	13.530**	0.026	15.350***	0.006	17.472***	0.000
**Wald chi2(5)**	47.040***	0.000	72.420***	0.000	77.630***	0.000
**Number of obs** = **630** **Number of groups** = **30** **Observation per group: Minimum** = **21** **Average** = **21.0** **Maximum** = **21**						

***, **, *indicates significance at 1 %, 5 % and 10 % levels. Source: Authors' calculations.

Results from the AMG model estimation, as indicated in Table 7, implied that human capital development (HCI), natural resource endowment (NRR), government expenditure (GFCE) and financial development (BM) were found to have a negative and statistically significant effect on economic sustainability in Sub-Saharan Africa. However, domestic investment (GCF) and the policy dummy (PD) variable, among the variables included in the economic sustainability model, had a positive and statistically significant effect on economic sustainability in the region.

Thus, in Sub-Saharan African countries, human capital development (HCI), measured based on years of schooling and returns to education, has a statistically significant negative effect on economic sustainability (ANNI). The result deviates from conventional economic theories, which state that the development of human capital has a positive impact on economic development. Furthermore, it differs from the majority of empirical research showing the positive relationship between the development of human capital and economic development [[26], [27], [28], [29], [30], [31], [32], [33]]. However, it aligns with some empirical research showing that human capital development has a long-term negative effect on Zimbabwe's economic growth [50].

Accordingly, the first main point of divergence from most empirical studies is the proxy used to measure human capital development. For instance, as a measure of human capital development, the majority of research used government spending on health and education as well as school enrolment rates. This study, however, made use of the human capital index (HCI), a more relevant measure, which is based on years of schooling and returns to education. Thus, unlike previous research that focuses on access to education dimensions, this study used HCI, which can clearly demonstrate the quality dimensions of education. Moreover, according to some research, the SSA region has been a major source of large-scale human capital flight and brain drain [2]. For instance, according to Ref. [17], one-third of the most educated people in SSA are thought to live outside of the region, primarily in Western Europe and North America, where earnings are higher. On the other hand, young graduates in the region were particularly vulnerable to severe unemployment [18]. These elements significantly affect the region's human capital development or return on investment in education, rendering educational investments counterproductive.

Additionally, there are several challenges to human capital development in Sub-Saharan Africa that make its contribution to economic sustainability negative. These include limited access to quality education, inadequate infrastructure, high levels of poverty, and political instability. Addressing these challenges requires inward-looking policies and coordinated efforts from governments, international organizations, and other stakeholders.

Moreover, natural resource endowment, measured by total natural resources rents as a percentage of GDP, was found to have a statistically significant negative effect on economic sustainability in Sub-Saharan Africa. This result is in line with empirical studies [51,52], which support the resource curse hypothesis put forward by Ref. [53], according to which resource-rich countries tend to grow more slowly than resource-poor ones. In Africa, particularly in Sub-Saharan Africa, natural resources has been observed to be a source of conflict and rampant corruption than serving development.

Further, government expenditure, measured by general government final consumption expenditure as a percentage of GDP, was found to have a statistically significant negative effect on economic sustainability in Sub-Saharan Africa. This finding is consistent with some empirical research, which claims that government spending in developing countries like SSA is mostly non-developmental, such as for defense, and debt servicing to crowd out private investment and economic development [54].

In addition, financial development, measured by broad money as a percentage of GDP, was found to have a statistically significant negative effect on economic sustainability in Sub-Saharan Africa. The result is in line with some empirical research, which demonstrates that financial development measured by domestic credit provided by the banking sector negatively affects economic development [55]. However, the result varied when a proxy like domestic credit to the private sector was used to measure financial development. But, the result is an indication of the under-development of the financial sector in Sub-Saharan Africa that impedes economic development in the region.

However, domestic investment, measured by gross capital formation, was found to have a statistically significant positive impact on economic sustainability in Sub-Saharan Africa. This finding is in line with the standard economics theory and earlier empirical research [56,57,26], which showed that domestic investment is a crucial driver of national income growth and sustainable economic development of countries. It has been discovered that domestic investment promotes domestic savings and has a multiplier effect on economic development.

Additionally, the policy dummy (PD) variable has a positive and statistically significant impact on the economic sustainability of Sub-Saharan African countries, suggesting that adjusted net national income has significantly increased in the region after 2015 or during the SDGs period as compared to the MDGs period. This finding is consistent with the sustainable development goals, which place a significant emphasis on the economic aspects of sustainability.

## 4 Conclusion and policy implications

Sustainable Development Goal number four (SDG-4) places a strong emphasis on quality education, which is crucial to human capital development. The importance of human capital development for sustainable economic development has thus risen to the top of the global policy agenda. However, the empirical literature on the topic has several limitations, including utilizing inappropriate measurement of human capital development and being unable to investigate the quality dimensions of education. This study used years of schooling and return on education to fill the gap and also took into account the role of institutional and political factors in its empirical estimation. This will help to minimize the bias in the regression estimates from omitted variables in earlier research. Further, in the empirical estimation of the model, the problems of cross-sectional dependence, slope heterogeneity, and co-integration are also accounted.

This study examined the effect of human capital development on economic sustainability in Sub-Saharan African countries using annual data ranging from 2000 to 2020, employing an augmented mean group (AMG) estimator. The empirical results demonstrate that human capital development negatively and significantly affects economic sustainability in Sub-Saharan African countries. Additionally, among other variables included in the model of economic sustainability, domestic investment (measured by gross capital formation) and the policy dummy have a positive and statistically significant effect on economic sustainability, while natural resource endowment, government expenditure and financial development have a negative and statistically significant effect on economic sustainability in Sub-Saharan Africa. As per the results, policy alternatives to mitigate the detrimental effects of human capital development on the economic sustainability of the region are discussed below.

The study recommends that governments and policymakers in Sub-Saharan African countries need to reconsider the whole educational architecture, including curriculum, strategy and financing. This will help them to understand the missing points somewhere and develop policies that fit the context. They should focus on improving access to quality education. This can be done through investments in infrastructure, teacher training, curriculum development, and the provision of scholarships and financial support to students and academic staff. Additionally, efforts should be made to enhance vocational training and technical skills development to meet the demands of the local labor market. This can help address the skills mismatch and promote entrepreneurship and innovation. Overall, human capital development is not negligible and essential for economic development in Sub-Saharan Africa. SSA countries can improve productivity, reduce poverty, and create sustainable economic development by investing in quality education and skills development.

To further achieve long-term economic development in SSA, boosting domestic investment or gross capital formation is essential. We learned from the policy dummy variable that policies like the sustainable development goals are essential for Sub-Saharan African countries to achieve economic sustainability; as a result, effective implementation of such policies is essential for the SDGs' remaining time periods in order to achieve the desired goals of economic sustainability and sustainable development.

As with most empirical investigations, this study is not free from limitations. First, the study is a regional-level analysis and might face aggregation problems. Therefore, country-specific investigations will be very important to strengthen the findings of this study. Further, due to data limitations in the public domain only 30 Sub-Saharan African countries are considered in the empirical analysis. Therefore, researchers with adequate research funds can collect sufficient country-level data from local government offices and will widen the scope of the study.

## Data availability statement

Data will be made available on request.

## CRediT authorship contribution statement

**Mulugeta Bekele:** Debelo, Writing - review & editing, Writing - original draft, Visualization, Validation, Software, Methodology, Investigation, Formal analysis, Data curation, Conceptualization. **Maria Sassi:** Writing - review & editing, Visualization, Validation, Supervision, Investigation, Formal analysis, Conceptualization. **Kedir Jemal:** Writing - review & editing, Visualization, Validation, Supervision, Investigation, Formal analysis, Conceptualization. **Beyan Ahmed:** Writing - review & editing, Visualization, Validation, Supervision, Investigation, Formal analysis, Conceptualization.

## Declaration of competing interest

The authors declare that they have no known competing financial interests or personal relationships that could have appeared to influence the work reported in this paper.

## References

[1] UN (2015).

[2] Egbulonu A.J., Bhattarai K. (2020). Determinants of capital flight: new panel evidence from sub-saharan Africa. Journal of Development Economics and Finance.

[3] Ramutsindela M., Mickler D. (2018).

[4] Jones L.E., Manuelli R.E. (1997). Endogenous growth theory: an introduction. J. Econ. Dynam. Control.

[5] Oghenekohwo J.E., Frank-Oputu E.A. (2017). Literacy education and sustainable development in developing societies. Int. J. Educ. Literacy Stud..

[6] Costantini V., Monni S. (2006). Environment, human development and economic growth. Ecol. Econ..

[7] Chikalipah S., Okafor G. (2019). Dynamic linkage between economic growth and human development: time series evidence from Nigeria. J. Int. Dev..

[8] Mahmood N., Wang Z., Hassan S.T. (2019). Renewable energy, economic growth, human capital, and CO2 emission: an empirical analysis. Environ. Sci. Pollut. Control Ser..

[9] Pata U.K., Ertugrul H.M. (2023). Do the Kyoto Protocol, geopolitical risks, human capital and natural resources affect the sustainability limit? A new environmental approach based on the LCC hypothesis. Resour. Pol..

[10] Saqib N., Usman M., Radulescu M., Sinisi C.I., Secara C.G., Tolea C. (2022). Revisiting EKC hypothesis in context of renewable energy, human development and moderating role of technological innovations in E-7 countries?. Front. Environ. Sci..

[11] Desha C., Robinson D., Sproul A. (2015). Working in partnership to develop engineering capability in energy efficiency. J. Clean. Prod..

[12] Saqib N., Sharif A., Razzaq A., Usman M. (2023). Integration of renewable energy and technological innovation in realizing environmental sustainability: the role of human capital in EKC framework. Environ. Sci. Pollut. Control Ser..

[13] Guloglu B., Caglar A.E., Pata U.K. (2023). Analyzing the determinants of the load capacity factor in OECD countries: evidence from advanced quantile panel data methods. Gondwana Res..

[14] Pata U.K., Isik C. (2021). Determinants of the load capacity factor in China: a novel dynamic ARDL approach for ecological footprint accounting. Resour. Pol..

[15] Pata U.K., Caglar A.E., Kartal M.T., Depren S.K. (2023). Evaluation of the role of clean energy technologies, human capital, urbanization, and income on the environmental quality in the United States. J. Clean. Prod..

[16] Saqib N., Duran I.A., Sharif I. (2022). Influence of energy structure, environmental regulations and human capital on ecological sustainability in EKC framework; evidence from MINT countries. Front. Environ. Sci..

[17] UNECA (2000). Regional Conference on Brain Drain and Capacity Building in Africa.

[18] Elder S., Koné K.S. (2014).

[19] Lewin K.M. (2020). Beyond business as usual: aid and financing education in Sub Saharan Africa. Int. J. Educ. Dev..

[20] WB (2022).

[21] Saqib N., Ozturk I., Usman M., Sharif A., Razzaq A. (2023). Pollution Haven or Halo? How European countries leverage FDI, energy, and human capital to alleviate their ecological footprint. Gondwana Res..

[22] Pata U.K., Tekin B., Özbay F. (2023). Empirical considerations on the reciprocal relationship between energy efficiency and leading variables: new evidence from OECD countries. Energy Build..

[23] Pata U.K., Caglar A.E. (2020). Investigating the EKC hypothesis with renewable energy consumption, human capital, globalization and trade openness for China: evidence from augmented ARDL approach with a structural break. Energy.

[24] Saqib N., Abbas S., Ozturk I., Murshed M., Tarczyńska-Łuniewska M., Mahtab-Alam M., Tarczyński W. (2023). Leveraging environmental ICT for carbon neutrality: analyzing the impact of financial development, renewable energy and human capital inTop polluting economies. Gondwana Res.

[25] Pata U.K., Erdogan S., Ozcan B. (2023). Evaluating the role of the share and intensity of renewable energy for sustainable development in Germany. J. Clean. Prod..

[26] Ohanyere C.P., Atueyi C.L., Angela I.O. (2019). Impact of human capital development on economic sustainability in Nigeria. International Academy Journal of Business Administration Annals.

[27] Ogundari K., Awokuse T. (2018). Human capital contribution to economic growth in Sub-Saharan Africa: does health status matter more than education?. Econ. Anal. Pol..

[28] Khan M. (2020). CO2 emissions and sustainable economic development: new evidence on the role of human capital. Sustain. Dev..

[29] Pelinescu E. (2015). The impact of human capital on economic growth. Procedia Econ. Finance.

[30] Sajoh A.A. (2021). The effect of human capital on economic growth in some Sub Sahara African countries (SSA). Am. J. Econ..

[31] Hakooma M.R., Seshamani V. (2017). The impact of human capital development on economic growth in Zambia: an econometric analysis. International Journal of Economics, Commerce and Management.

[32] Ali S., Alam K.J., Noor T. (2016). An econometric analysis of human capital development and economic growth in Bangladesh. J. Econ. Sustain. Dev..

[33] Ekperiware M.C., Olatayo T.O., Egbetokun A.A. (2017).

[34] Lin X., Ahmed Z., Jiang X., Pata U.K. (2023). Evaluating the link between innovative human capital and regional sustainable development: empirical evidence from China. Environ. Sci. Pollut. Control Ser..

[35] Johnson A.O. (2011). Human capital development and economic growth in Nigeria. Eur. J. Bus. Manag..

[36] Arestis P., Baddeley M., McCombie J.S., Elgar E. (2007).

[37] Barbier E.B. (1987). The concept of sustainable economic development. Environ. Conserv..

[38] Ahmed M.M., Shimada K. (2019). The effect of renewable energy consumption on sustainable economic development: evidence from emerging and developing economies. Energies.

[39] Swain R.B., Yang-Wallentin F. (2020). Achieving sustainable development goals: predicaments and strategies. Int. J. Sustain. Dev. World Ecol..

[40] Vasylieva T., Lyulyov O., Bilan Y., Streimikiene D. (2019). Sustainable economic development and greenhouse gas emissions: the dynamic impact of renewable energy consumption, GDP, and corruption. Energies.

[41] Pesaran M.H. (2004).

[42] Pesaran M.H., Yamagata T. (2008). Testing slope homogeneity in large panels. J. Econom..

[43] Bilgili F., Ulucak R. (2018). Is there deterministic, stochastic, and/or club convergence in ecological footprint indicator among G20 countries?. Environ. Sci. Pollut. Control Ser..

[44] Breusch T., Pagan A. (1980). The LM test and its application to model specification in econometrics. Rev. Econ. Stud..

[45] Westerlund J. (2007). Testing for error correction in panel data. Oxf. Bull. Econ. Stat..

[46] Pesaran M.H., Smith R.P. (1995). Estimating long-run relationships from dynamic heterogeneous panels. J. Econom..

[47] Paramati S.R., Mo D., Gupta R. (2017). The effects of stock market growth and renewable energy use on CO2 emissions: evidence from G20 countries. Energy Econ..

[48] Dumitrescu E., Hurlin C. (2012). Testing for Granger non-causality in heterogeneous panels. Econ. Modell..

[49] Eberhardt M., Bond S. (2009).

[50] Abel S., Mhaka N., Roux P.L. (2019). Human capital development and economic growth nexus in Zimbabwe. South. Afr. Bus. Rev..

[51] Satti S.L., Farooq A., Loganathan N., Shahbaz M. (2014). Empirical evidence on the resource curse hypothesis in oil abundant economy. Econ. Modell..

[52] Mulugeta B., Eyerusalem M. (2020). Natural resource endowment and sustainable development linkage in Ethiopia. Journal of Resources Development and Management.

[53] Sachs J.D., Warner A.M. (2001). Natural resources and economic development: the curse of natural resources. Eur. Econ. Rev..

[54] Hussain A., Mohammad S.D., Akram K., Lal I. (2009). Effectiveness of government expenditure crowding-in or crowding-out: empirical evidence in case of Pakistan. Eur. J. Econ. Finance Adm. Sci..

[55] Hassan M.K., Sanchez B., Yu J. (2011). Financial development and economic growth: new evidence from panel data. Q. Rev. Econ. Finance.

[56] Shabbir M.S., Bashir M., Abbasi H.M., Yahya G., Abbasi B.A. (2021). Effect of domestic and foreign private investment on economic growth of Pakistan. Transnational Corporations Review.

[57] Abu N., Karim M.Z. (2016). The relationships between foreign direct investment, domestic savings, domestic investment, and economic growth: the case of sub-saharan Africa. Soc. Econ..

